# A unique case of Bloom syndrome with a combination of genetic hits: A lesson from trio‑based exome sequencing: A case report

**DOI:** 10.3892/mmr.2023.12997

**Published:** 2023-04-12

**Authors:** Marketa Wayhelova, Vladimira Vallova, Petr Broz, Aneta Mikulasova, Dominika Machackova, Hana Dynkova Filkova, Jan Smetana, Alena Takacsova, Renata Gaillyova, Petr Kuglik

**Affiliations:** 1 Department of Experimental Biology, Faculty of Science, Masaryk University, 61137 Brno, Czech Republic; 2 Laboratory of Cytogenomics, Centre of Molecular Biology and Genetics, Department of Internal Medicine, Hematology and Oncology, University Hospital Brno, 62500 Brno, Czech Republic; 3 Department of Biology and Medical Genetics, 2nd Faculty of Medicine, Charles University, Prague and Faculty Hospital Motol, 15006 Prague, Czech Republic; 4 Biosciences Institute, Newcastle University, Newcastle upon Tyne NE2 4HH, United Kingdom; 5 Department of Medical Genetics and Genomics, University Hospital Brno, 62500 Brno, Czech Republic

**Keywords:** exome sequencing, *BLM* gene, Bloom syndrome, cancer-predisposing syndrome, copy-number neutral loss of heterozygosity

## Abstract

Pathogenic variants affecting the *BLM* gene are responsible for the manifestation of extremely rare cancer-predisposing Bloom syndrome. The present study reports on a case of an infant with a congenital hypotrophy, short stature and abnormal facial appearance. Initially she was examined using a routine molecular diagnostic algorithm, including the cytogenetic analysis of her karyotype, microarray analysis and methylation-specific MLPA, however, she remained undiagnosed on a molecular level. Therefore, she and her parents were enrolled in the project of trio-based exome sequencing (ES) using Human Core Exome kit. She was revealed as a carrier of an extremely rare combination of causative sequence variants altering the *BLM* gene (NM_000057.4), c.1642C>T and c.2207_2212delinsTAGATTC in the compound heterozygosity, resulting in a diagnosis of Bloom syndrome. Simultaneously, a mosaic loss of heterozygosity of chromosome 11p was detected and then confirmed as a borderline imprinting center 1 hypermethylation on chromosome 11p15. The diagnosis of Bloom syndrome and mosaic copy-number neutral loss of heterozygosity of chromosome 11p increases a lifetime risk to develop any types of malignancy. This case demonstrates the trio-based ES as a complex approach for the molecular diagnostics of rare pediatric diseases.

## Introduction

The clinical utility of exome sequencing (ES) has fundamentally deepened our knowledge of the molecular basis of neurodevelopmental and other rare pediatric disorders. Current data point to a growing number of genes and leading mechanisms implicated in rare Mendelian disorders, requiring exome or genome sequencing and additional diagnostics approaches on transcriptional level such as RNA sequencing and epigenome analysis (1,2).

The *BLM* gene (15q26.1), also termed *RECQL3*, encodes an intracellular nuclear protein belonging to the RecQ family of 3′ to 5′ DNA helicases. It is a crucial component of complex processes of the cell cycle regulation and DNA repair which maintain the genome stability (3). At present, the ClinVar database (https://www.ncbi.nlm.nih.gov/clinvar) summarizes >350 pathogenic and/or likely pathogenic variants altering the primary sequence of the *BLM* gene and resulting in the typical phenotype of Bloom syndrome (retrieved March 27, 2023). The affected individuals manifest a microcephaly, severe growth restriction, slender physique, general hypotrophy and abnormal facial appearance including long narrow face, small lower jaw and prominent nose and ears (4). They often suffer from immune deficiency and insulin resistance. They have a dramatically increased susceptibility for early-onset malignancies and lifetime risk to develop any type of them due to the impaired functioning of DNA repair machinery (4). The absence of the functional BLM RecQ-like helicase results in a chromosomal instability, excessive number of chromosomal breaks with consequent homologous recombination leading to sister chromatid exchanges (SCE) (5).

## Case report

### Case presentation

The proband was a two-year-old girl (born 2020) with an initial clinical diagnosis of intrauterine growth restriction, postnatal growth deficiency, general hypotrophy and abnormal facial appearance. She is an only offspring of non-consanguineous couple (mother born in 1993, father born in 1990) of Caucasian origin. They are clinically healthy, both with family history lacking consanguinity or any abnormal phenotypic features with a relevance to that of the proband. The prenatal biochemical and ultrasound screening tests in the first trimester were evaluated as normal. Since the 24th week of pregnancy she had been monitored due to the intrauterine growth restriction and amniotic fluid deficiency. She was delivered prematurely after 36 weeks and 3 days of pregnancy (1,570 g/39 cm). She had been under the medical supervision for 5 weeks, including 24-h phototherapy due to the icterus neonatorum and three days in the incubator. She had been breast-fed for four months. She has been doing Vojta therapy (6) for one year (between February 2020 and February 2021). She was hospitalized due to a failure to thrive (not gaining weight) in July 2020. She underwent the clinical genetics evaluation at the age of 6 (July 2020) and 22 months (November 2021). The last medical observation at 30 months (June 2022) summarized the short proportionate stature (75.4 cm; Z-score −3.6) with a general paucity of subcutaneous fat (body weight 6,800 g; Z-score 0), microcephaly and dolichocephaly (head circumference 41 cm), normal intellect, speech and motor development, persistent failure to thrive and difficulty in feeding. She manifested distinct facial abnormalities (triangle face, bilateral epicanthus, mild hypertelorism, narrow long nose, anteverted nostrils and long philtrum), square-shaped palms and mild 5th finger clinodactyly. A mild facial symmetric erythema had been observed, as well as two Café-au-lait spots on the left thigh. The immunologic examination has shown mildly decreased levels of B lymphocytes and immunoglobulins (IgA, IgG and IgM). She is under the preventive medical surveillance at the Clinic of Children's Oncology (University Hospital Brno, Czech Republic). The recent preventive examination using magnetic resonance excluded organomegaly, the levels of tumor markers (alpha-fetoprotein, beta-human chorionic gonadotropin, neuron-specific enolase) were assessed as normal. She regularly undergoes medical examinations and counselling at specialized clinics (including gastroenterology, immunology, pediatrics and endocrinology). All procedures performed involving human participants were in accordance with the ethical standards of the institutional and/or national research committee and with the 1964 Helsinki declaration and its later amendments or comparable ethical standards. Approval was obtained from the Research Ethics Committee of Masaryk University (approval no. EKV-2019-056) and Ethics Committee of University Hospital Brno (approval no. 10-120619/EK). Written informed consent was obtained from the parents of the patient (proband) before the procedure of genetic analyses.

## Materials and methods

### Cytogenetic and molecular cytogenetic analysis

The peripheral blood samples of proband and her parents were obtained for cytogenetic analysis of karyotype and for DNA extraction [using the MagNA Pure 96 System (Roche Diagnostics, Ltd.) according to the manufacturer's recommendations] for molecular cytogenetic/genetic analyses. The cytogenetic analysis of proband's karyotype was performed using the routine G-banding procedure by Giemsa-Romanowski staining (7). The whole-genome screening of submicroscopic copy-number variations (CNVs) and copy-number neutral losses of heterozygosity (cnnLOH) was performed using the oligonucleotide-based microarray platform SurePrint G3 ISCA v2 CGH+SNP 4X180K Microarray following the manufacturer's recommendations (Agilent Technologies, Inc.). Microarray data were extracted and processed to the CGH+SNP profile visualization using the Agilent Cytogenomics 4.0.3. software (Agilent Technologies, Inc.). CNVs were called using the ADM-2 algorithm with the settings of at least three neighboring probes in a genomic region, a minimal size of 100 kb and minimal absolute log2 ratio 0.25. The regions of cnnLOH were evaluated at ~10 Mb resolution (LOH score ≥6) across the entire genome.

### Relative quantification using quantitative (q)PCR

Relative quantification using qPCR was then performed to verify the 15q11.2 microduplication, with two pairs of DNA primers (Integrated DNA Technologies, Inc.) which were designed to prime the DNA sequence within and outside the targeted CNV. The reaction mixtures were prepared with PowerSYBR™ Green PCR MasterMix (Thermo Fisher Scientific, Inc.) and run on a Light Cycler^®^ 480 Real-Time PCR System (Roche Diagnostics, Ltd.) according to the manufacturer's recommendation. The thermocycling conditions were as follows: 95°C/10 min (initial denaturation), 40 cycles of 95°C/15 sec, 60°C/1 min (with data acquisition in every cycle), denaturation at 95°C/15 sec, melting curve generation from 60°C/1 min to 95°C with continuous data acquisition, and then cooling. The relative quantification was performed using the formula 2^−ΔΔCq^ with the threshold R-value <0.7 for DNA loss and >1.3 for DNA gain in the region of interest relatively to the reference gene *ERH* (8). The sequences of the primers are listed in Table SI (Sheet 1).

### Methylation-specific MLPA

The methylation-specific multiplex ligation-dependent probe amplification (MS-MLPA) analysis was performed using the SALSA^®^ MLPA^®^ Probemix ME030 BWS/RSS (MRC Holland). The data were analyzed using the Coffalyser.Net software according to the manufacturer's recommendations (MRC Holland).

### Exome sequencing

High-quality of genomic DNA samples (~200 ng) were used for the library preparation with the Human Core Exome kit, which provides 33 Mb CCDS coverage with 99% ClinVar variants coverage, with spiked-in Human RefSeq panel (Twist Bioscience) and custom spiked-in probes for mitochondrial DNA. The DNA libraries were then sequenced on Illumina NovaSeq 6000 (Illumina, Inc.). All steps were performed as a commercially available service (Institute of Applied Biotechnologies A.S.) according to the manufacturer's recommendations.

### Bioinformatic processing of ES data

Raw sequencing data were processed to obtain sequence variants, CNVs and cnnLOH, as described previously (9). Briefly, the quality control (QC) was performed using the FastQC v0.11.9 (https://www.bioinformatics.babraham.ac.uk/projects/fastqc/). The low-quality reads and adapter contamination trimming was performed by the fastp v0.20.1 (10). The remaining reads were aligned to the reference human genome hg38/GRCh38 primary assembly by a software package BWA v0.7.17-r1188 (11) with default parameters following by marking duplicate reads and fix mate information using Picard tools 2.27.5 (http://broadinstitute.github.io/picard/). QC steps and the coverage were checked using the in-house software Genovesa (developed by Bioxsys, s.r.o; http://www.bioxsys.eu/#/genovesa). Single-nucleotide variants (SNVs) and insertion/deletion variants (indels) were called using the VarScan v2.4.4 (with parameters: Min-coverage, 20; min-var-freq, 0.1; P-value, 0.5; min-avg-qual, 10) (12). The variant calling process is based on a Fisher's Exact test, a statistical test procedure that calculates an exact probability value for the relationship between two dichotomous variables, as found in a two by two crosstable. The program calculates the difference between read counts supporting reference and variant alleles with P-value threshold 0.05. Only SNVs and indels passing the quality filter (a minimal quality of coverage ≥20X, base quality ≥10, mapping quality ≥5) and with an alternative allele frequency ≥10% per sample, P-value (Fisher exact test) <0.05 were included for further variant filtering.

CNVs were called using two different bioinformatics pipelines. The first approach was based on the depth calculation and normalization using the R software v3.6.0 (https://www.r-project.org/) in covered exons (13). Those exons which failed the mapability criteria (lower than 0.75 defined using 35-mer mapability score from UCSC genome browser) were excluded from the analysis. The read depth coverage base line was created using ≥6 samples and then the algorithm compared each sample to each. The ratio of expected reads to real number of reads was calculated to estimate a gain or loss in any specific locus defined by target.

The second approach was a custom pipeline CNVRobot v3.5 (https://github.com/AnetaMikulasova/CNVRobot). Briefly, GATK tools v4.2.4.1 (Broad Institute) were used for the processing of bam files and data denoising. CNVs and losses of heterozygosity (LOH) were called using a custom R-based segmentation and filtered by parameters as follows: CNVs; ≥50 bp and two intervals; ≤-0.5 Log2 Ratio (L2R) for losses and >0.3 L2R for gains. LOH; ≥5 Mb and 10,000 intervals. Unaffected unrelated sex-matching individuals (31 males and 31 females) served as controls for data denoising.

### Variant prioritization and classification

The filtering conditions were set to search for only those sequence variants with ≥20% frequency (% of reads with the variant) in the proband, the impact ‘moderate’ or ‘high’ based on the Ensembl Variant Effect Predictor (v105) (14), allele frequencies ≤5% in the non-Finnish European population (for known variants with annotations) or with an unknown allele frequency (for novel variants). Then Locus Reference Genomics (LRG) or Canonical Transcripts were selected for reporting of clinically relevant sequence variants. Only variants with a ‘pathogenic’ and ‘likely pathogenic’ clinical impact based on the current version of the ClinVar database (15) or candidate novel variants in OMIM ‘morbid’ genes were considered for further analysis. The pathogenicity of novel variants in OMIM ‘disease-causing’ genes was evaluated using the VarSome engine including the current ACMG classification (16). The general information about genes were obtained from the OMIM database (17). Only clinically relevant causative variants were then reported to clinicians. CNVs were filtered according to technical cut-offs: reads ratios ≤0.7 for losses, ≥1.3 for gains; log2 ratios ≤-0.5 for losses and ≥0.35 for gains. Then CNVs encompassing OMIM ‘morbid’ or candidate ‘morbid’ genes or CNVs classified as pathogenic or likely pathogenic in the dbVar database (dbVar Genome Browser; v2.8) were prioritized for further analysis (18). The presence of cnnLOH was assessed after the manual curation to evaluate long stretches of homozygous genotypes from the ES-based sequence variants analysis and CGH+SNP microarray analysis.

### Sanger sequencing

The presence of pathogenic sequence variants was then confirmed using Sanger sequencing with two pairs of custom primers: forward primer BLM_1_F 5′-CTGGGCTGAAACACCAAGAC-3′, reverse primer BLM_1_R 5′-GCAGCTGTGGAAGATTTGCT-3′ and forward primer BLM_2_F 5′-GCCCTGCCTGAGTTATGCT-3′, reverse primer BLM_2_R 5′-CCATTTGGGGTTTCTGGATGA-3′ (Integrated DNA Technologies) as described in detail elsewhere (6). The sequencing reactions were run on the capillary sequencer ABI 3130 (Thermo Fisher Scientific, Inc.) with further analyses using the freeware FinchTV (Geospiza, Inc.).

## Results

### Quality control of technical parameters of ES

On average, >90 million unique reads were mapped to the reference genome GRCh38/hg38 primary assembly: ~99% of targeted bases were covered to at least 30X and the median target coverage was higher than 100X, reaching an essential quality for a reliable evaluation and interpretation of ES outputs in the routine molecular genetic diagnostics. The average proportion of flagged PCR duplicates was only 16% and the average uniformity assessed from all samples involved in a research project reached 1.37 which is a good assumption for CNV analysis. The values of technical parameters and QC metrics are available in the Table SII.

### Cytogenetic analysis and molecular cytogenetic analysis

The cytogenetic analysis of the proband's karyotype was performed with a result of normal female karyotype 46,XX. She immediately underwent the microarray analysis using the oligonucleotide-based CGH+SNP microarray with a finding of a recurrent 15q11.2 microduplication (BP1-BP2), classified as likely benign (19). The parental testing by qPCR proved its familial origin as it was confirmed in her father and paternal grandfather. The outputs are available in Table SI (Sheet 2). The MS-MLPA analysis was performed with the SALSA^®^ MLPA^®^ Probemix ME030 BWS/RSS (MRC Holland) due to the severe growth restriction as a typical phenotypic manifestation of Silver-Russell (SRS) or Silver-Russell syndrome-like phenotype (SRS-like). The outputs proved the borderline hypermethylation *H19* IC1 locus (0.73-0.8) on chromosome 11p15. However, this result did not match the SRS or SRS-like phenotype.

### Trio-based ES

**Figure 1.**
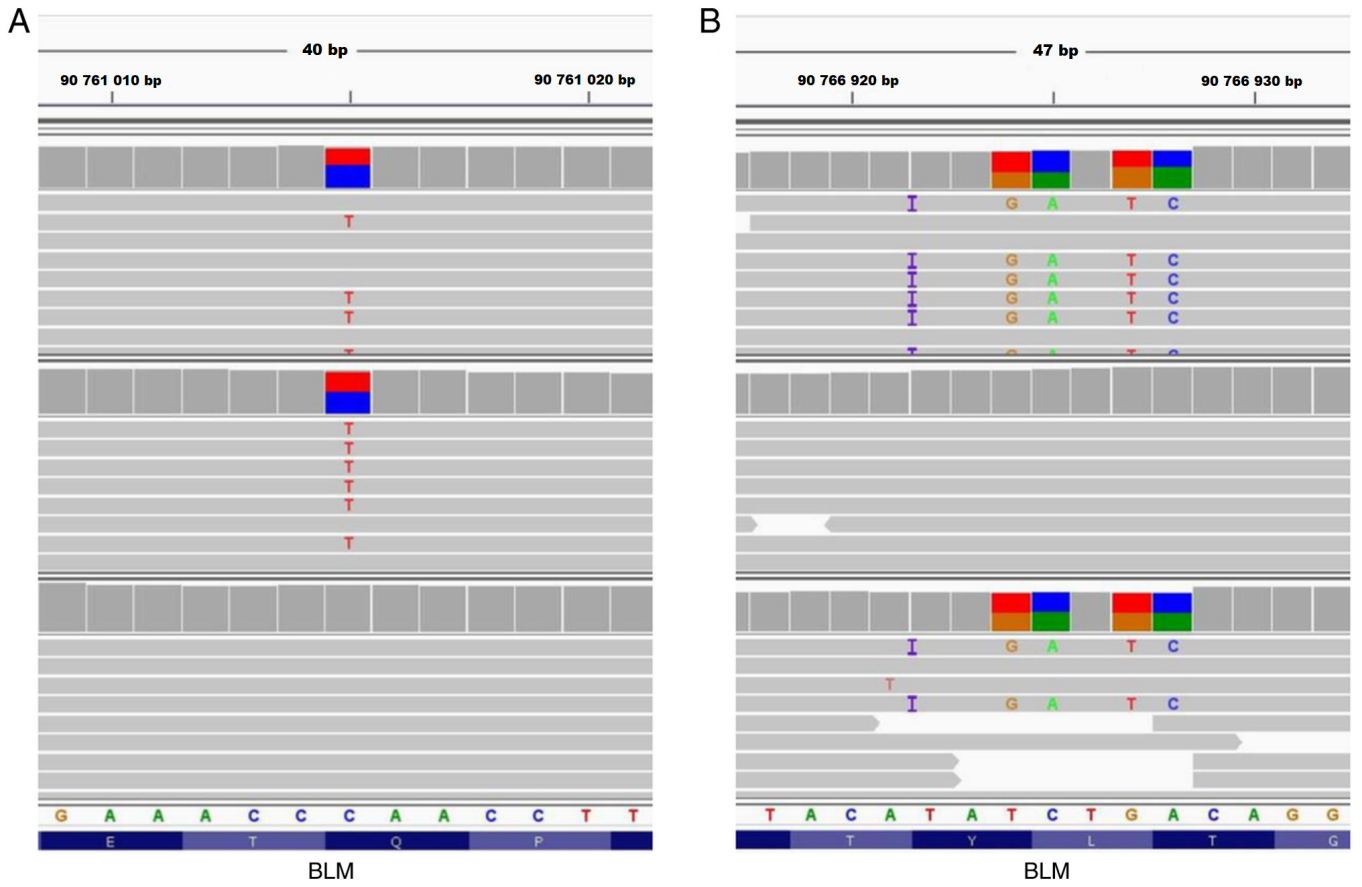
Pathogenic *BLM* gene variants detected by ES. (A) Visualization of the substitution in the *BLM* gene, NM_000057.4:c.1642C>T detected by ES. The panels with horizontal grey lines represent mapped reads in the proband, her mother and father (from top to bottom). The substitution C>T in proband and her mother located in the middle part of panels was detected in ~43% of mapped reads [visualized in IGV software v2.8.13; https://software.broadinstitute.org/software/igv/, (43)]. (B) The visualization of the deletion-insertion in the *BLM* gene, NM_000057.4:c.2207_2212delinsTAGATTC detected by ES. The panels with horizontal grey lines represent single mapped reads in the proband, her mother and father (from top to bottom). The ATCTGA deletion replaced by TAGATTC insertion in proband and her father located in the middle part of panels was detected in ~47% of mapped reads (visualized in IGV software v2.8.13). The colored blocks represent the (heterozygous) substitution. ES, exome sequencing.

As the routine molecular genetic testing (cytogenetic analysis and CGH+SNP microarray analysis) was negative, the proband and her unaffected parents were enrolled for trio-based ES. After the variant filtering and their evaluation using the VarSome engine, medical and scientific literature and databases two clinically relevant variants affecting the *BLM* gene, c.1642C>T and c.2207_2212delinsTAGATTC (NM_000057.4), were detected. Their compound heterozygosity was assessed for the substitution c.1642C>T of maternal origin and deletion-insertion c.2207_2212delinsTAGATTC of paternal origin (Fig. 1). These findings were then verified by Sanger sequencing. No incidental reportable findings affecting the ‘medically-actionable’ genes based on the ACMG recommendation were detected (20).

The variant c.1642C>T in the exon 7 is a well-known variant (rs200389141) with a non-Finnish European allele frequency ~0.03-0.04% but rising to a carrier frequency of ~0.1% in the Eastern Slavic population, which is the highest observed frequency (4). Due to the substitution c.1642C>T, the premature termination codon for a nonsense variant p.(Gln548Ter) is predicted, however, the aberrant transcripts are likely to be degraded by a nonsense-mediated mRNA decay (NMD) pathway.

By contrast, the deletion-insertion c.2207_2212delinsTAGATTC variant (rs113993962) in the exon 10 of the *BLM* gene predominates as a founder allele in Ashkenazi Jewish population and their descendants (BLM^Ash^), reaching an estimated carrier frequency ~1% (21). It alters the DNA sequence of the exon 10, which is translated to a part of the helicase ATP-binding domain. The production of the truncated protein due to the deletion-insertion c.2207_2212TAGATTC, p.(Tyr736LeufsTer5) is prevented by the NMD pathway based on *in silico* prediction tools.

**Figure 2.**
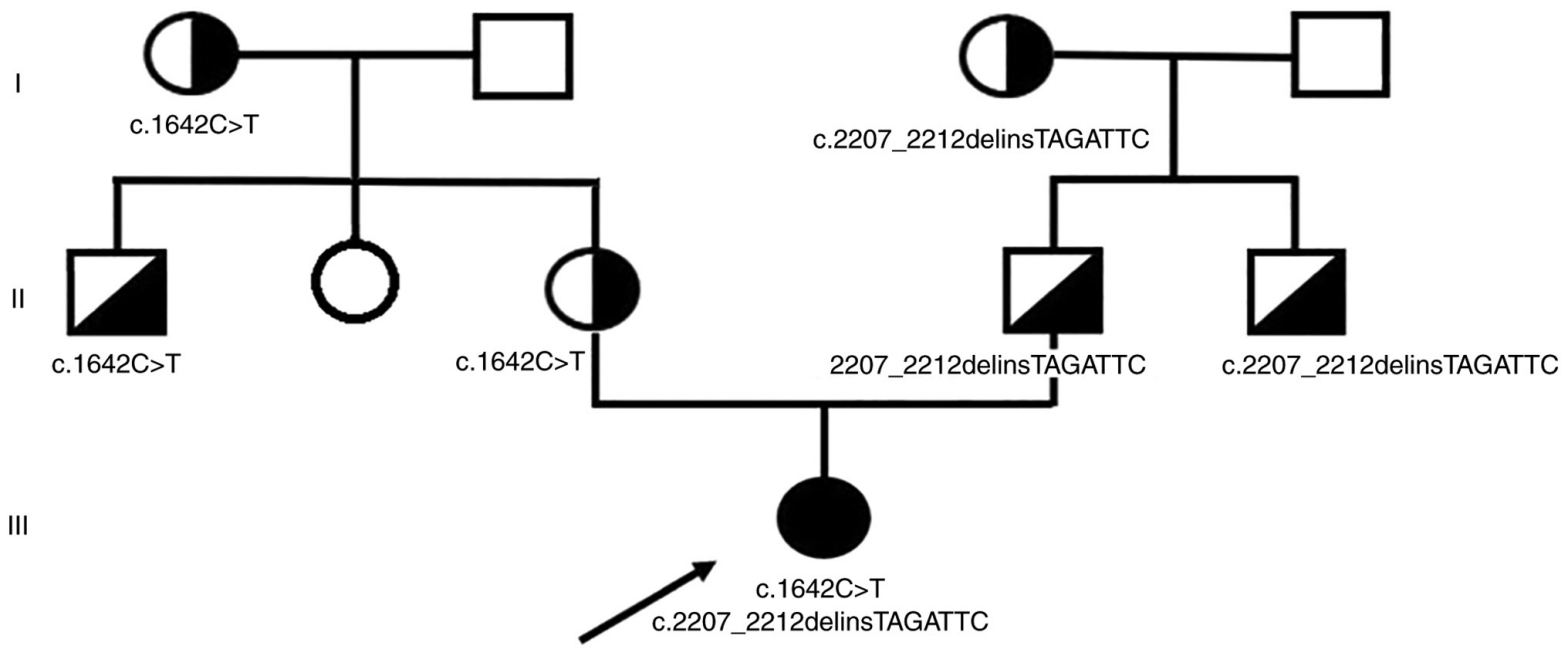
Pedigree of the family with the familial transmission of the causative *BLM* gene variants.

**Figure 3.**
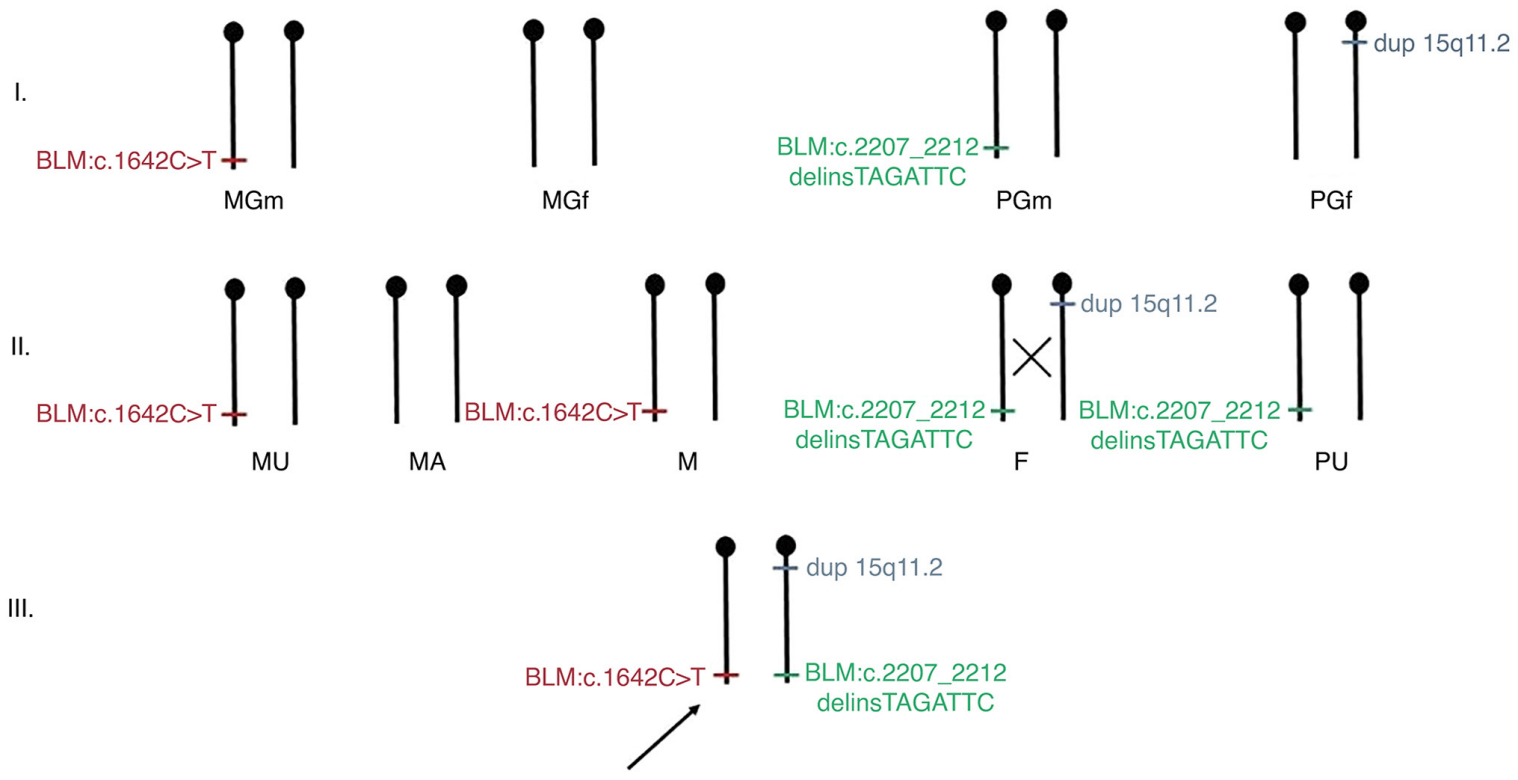
Schematic visualization of the family with the segregation of the causative BLM gene variants and 15q11.2 microduplication. The variant segregation analysis suggested the meiotic crossing-over between 15q11.2 microduplication and the BLM gene during the spermatogenesis of proband's father. MGm – maternal grandmother, MGf – maternal grandfather, PGm – paternal grandmother, PGf – paternal grandfather, MU – maternal uncle, MA – maternal aunt, M – mother, F – father, PU – paternal uncle.

The outputs of *in silico* prediction tools, ACMG classification criteria and database records and literature data confirming the pathogenicity of the *BLM* variants are summarized in Table SIII (Sheets 1 and 2). The targeted analyses for the identification of c.1642C>T and c.2207_2212delinsTAGATTC variants using Sanger sequencing and the 15q11.2 microduplication (BP1-BP2) using qPCR were performed in maternal and paternal relatives (Fig. 2). The pathogenicity of the 15q11.2 microduplication has been discussed elsewhere and the current approach is to classify it as benign and not to report it (19). However, in our case report the 15q11.2 microduplication incidentally serves as a marker of a probable meiotic crossing-over between it the *BLM* gene (15q26.1) during the spermatogenesis of proband's father (Fig. 3).

**Figure 4.**
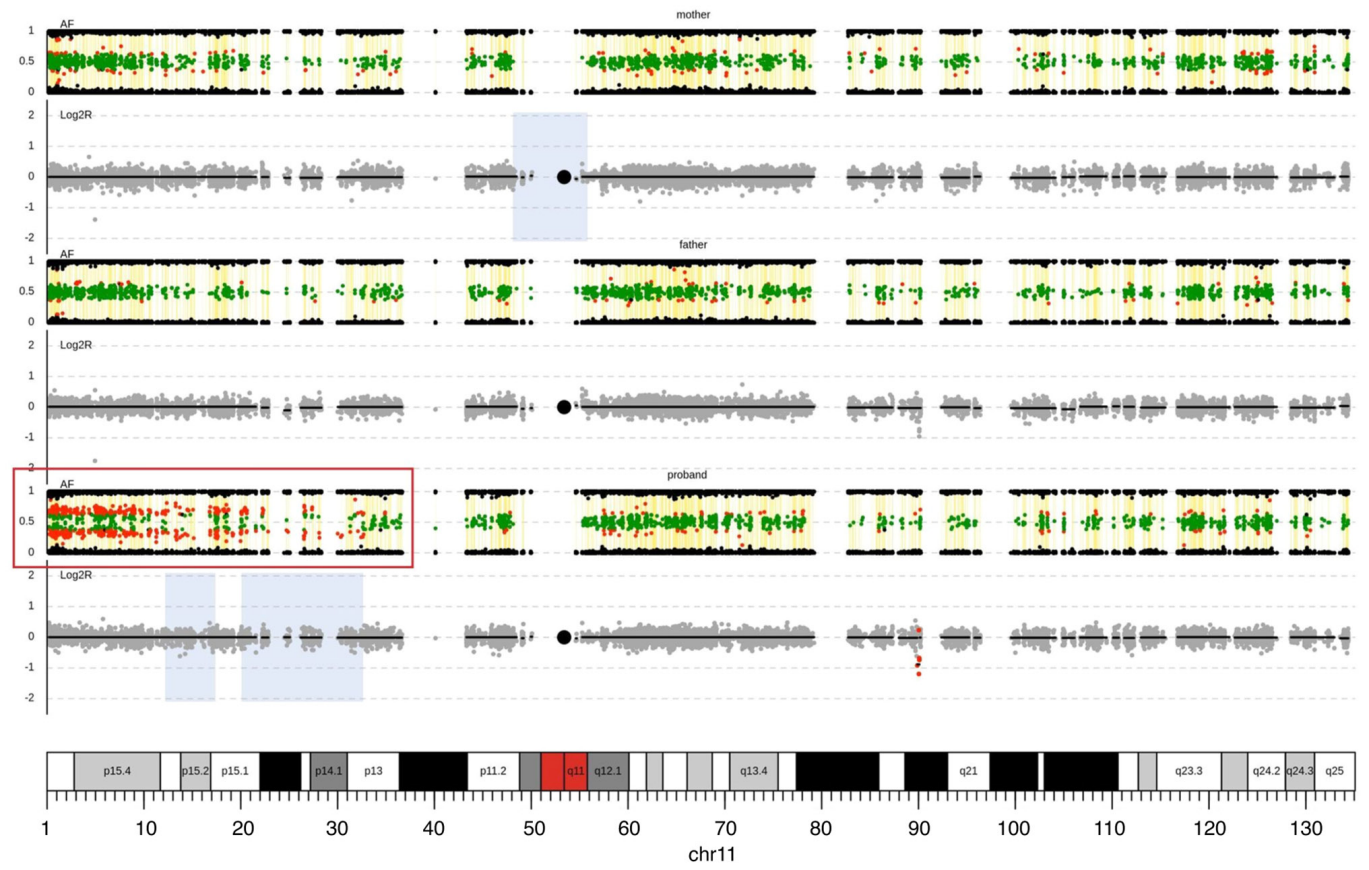
Mosaic cnnLOH of the chromosome 11p detected by ES. The panel shows profiles of chromosome 11 in mother, father and proband (from top to bottom). Log2 Ratio (L2R) tracks represent the copy-number profiles. Grey dots mark sequencing targets without any copy-number abnormality. Red and green dots indicate presence of losses and gains, respectively. In the AF track, black dots represent SNPs with a single allele detected in the sequencing reads and green and red dots highlight SNPs with two alleles found in ~1:1 and another ratio, respectively. The mosaic cnnLOH at chromosome 11p in proband is visualized as allele frequency imbalances for heterozygosity (expected average allele frequency for heterozygosity 0.5 vs. identified allele frequency 0.7-0.8) in the AF track (in the red frame in proband's profile). cnnLOH, copy-number neutral losses of heterozygosity; ES, exome sequencing; AF, allele frequency; SNPs, single nucleotide polymorphisms.

The simultaneous CNV and cnnLOH analysis from ES data was performed, with a verification of the 15q11.2 microduplication in the proband and her father (Fig. S1), which was initially identified by the microarray analysis. Moreover, an additional cnnLOH analysis from ES data uncovered the somatic mosaic cnnLOH of chromosome 11p which agrees with the output of MS-MLPA analysis; the borderline hypermethylation *H19* IC1 locus (Fig. 4). The mosaic cnnLOH of chromosome11p is then evident due to the imbalances of the allelic ratios for heterozygous SNVs on chromosome 11p in proband.

## Discussion

The present case report provided a proof of a wide diagnostic utility of trio-based ES in the molecular genetic diagnostics of pediatric rare diseases. The simultaneous detection of sequence variants, CNVs and cnnLOH identified a unique co-occurrence of compound heterozygous rare pathogenic variants of the *BLM* gene and mosaic cnnLOH on chromosome 11p. These two clinically relevant genetic entities represent important medically actionable issues for the utility of personalized medicine.

The *BLM* gene is located on 15q26.1 chromosome and provides instruction for ATP-dependent RecQ helicase. It is a component of BRCA1-associated genome surveillance complex, which plays multiple roles in the DNA damage response to maintain the genomic stability (4). It unwinds single-strand (ss)- and double-strand (ds)DNA in a 3′ to 5′ direction, participates in DNA replication and in the repair of double-strand breaks. The BLM RecQ-like helicase prevents SCE events, therefore their identification in metaphases is a cytogenetic marker of Bloom syndrome and other syndromes of the chromosomal instability (22).

The *BLM* gene structure is divided into several domains providing the key effector functions, which disruptions lead to an increased cellular sensitivity for DNA damage. Therefore, the *BLM* gene is highly expressed in rapidly proliferating cells and is cell-cycle regulated, reaching the highest level in the late S and G_2_ phases (4).

The pathogenic variants including missense and truncating sequence variants and intragenic deletions of the *BLM* gene have been confirmed as the molecular genetic cause of Bloom syndrome, an autosomal recessive disorder affecting multiple body tissues and organ systems. The Bloom Syndrome registry (23) and other medical literature provide details about ~300 reported cases of individuals with Bloom syndrome since 1954, when the first case of Bloom syndrome was documented. To the best of the authors' knowledge, the *BLM* gene is the only gene responsible for the clinical manifestation of Bloom syndrome. However, there are three other genes *RMI1, RMI2* and *TOP3A* encoding proteins, and which form a complex with the BLM RecQ-like helicase. Their pathogenic variants can cause a milder phenotype than is observed in individuals with Bloom syndrome (‘Bloom syndrome-like’ phenotype) (24). Therefore, it is suggested that individuals in the Bloom syndrome registry lacking molecular diagnosis may have causative variants in other genes as *RMI1, RMI2*, or *TOP3A* genes with the overlapping phenotypic manifestation. Recently, novel deep intronic variant leading to a pseudo-exon activation has been detected using RNA-based long-range PCR in an individual with Bloom syndrome and only one causative variant in the *BLM* gene which was detected in the previous analysis (25). Therefore, novel approaches including genome sequencing or transcriptome analysis may complete the molecular diagnosis of Bloom syndrome in those individuals with the phenotypic manifestation of Bloom syndrome in which only one causative variant in the *BLM* gene was detected using the sequencing analysis of its coding region (23,25). Moreover, a study using single-cell transcriptomic profiling uncover an altered transcriptional profile and suggested novel links between BLM helicase dysfunction and aberrant transcription of condensin complexes genes (26).

To the best of the authors' knowledge, >350 different causative variants of the *BLM* gene have been identified as causative, including some founder variants with a higher frequency in certain populations or ethnic groups (15,23).

The recurrent variant c.1642C>T is enriched in the Eastern Europe population of the Slavic origin, in which 0.2-0.6% individuals are its carriers. Only a few patients with Bloom syndrome carrying homozygous c.1642C>T variant are described in a scientific literature, probably due to the incomplete phenotypic manifestation lacking the presence of a typical UV exposure-induced facial erythema (27,28). It raises the hypothesis of the underdiagnosis of Bloom syndrome at the clinical and molecular level in this population. The variant affects the protein region which interacts with the scaffolding protein involved in DNA repair (SPIDR). SPIDR interconnects BLM and RAD51 proteins and targets them to sites of DNA damage (29). Certain studies show the association between the c.1642C>T variant and an increased risk for breast cancer (0.5–1% breast cancer in Slavic population) (30,31).

The recurrent variant c.2207_2212delinsTAGATTC has been observed as a founder allele in Ashkenazi Jews and their descendants with a frequency of 1%, therefore the term *BLM*^Ash^ is widely used. Due to the migration and founder effect, it has been established independently in different regions worldwide. The estimated prevalence of Bloom syndrome in the Ashkenazi Jewish population reaches ~1:48 000, but it occurs extremely rarely in the general population (32). The carriers of the *BLM*^Ash^ allele may come up against an increased risk of developing any type of malignancy; however, no significant association has been observed so far.

Our proband is a compound heterozygote for these two founder alleles, maternally inherited c.1642C>T and paternally inherited c.2207_2212delinsTAGATTC. The frequencies of both pathogenic variants of BLM gene are rare in the general Caucasian population, however, they occur in higher frequencies in certain populations (c.1642C>T in Slavic population and c.2207_2212delinsTAGATTC in the population of Ashkenazi Jews and their descendants) (4,21). The compound heterozygosity for these variants is extremely rare which is documented by a rare frequency of Bloom syndrome in the general Caucasian population. The worldwide incidence of Bloom syndrome due to biallelic causative variants in the *BLM* gene is unknown, ~300 cases have been reported so far in databases and in the medical literature. Although its prevalence in the population of Ashkenazi Jews is estimated to be ~1:48,000, only ~1/3 of individuals with Bloom syndrome due to the causative variants in the *BLM* gene are of Ashkenazi Jewish descent.

Another documented case of an infant carrying these two causative *BLM* gene variants in the compound heterozygosity has been published recently (33). He was diagnosed with Bloom syndrome at the age of 9 years, but he developed an infantile fibrosarcoma at 6 months. His case demonstrates a dramatically increased risk for childhood malignancies in individuals with Bloom syndrome and points out the importance of multidisciplinary medical long-term follow up.

In addition, our proband is a carrier of a mosaic cnnLOH of chromosome 11p corresponding to the borderline imprinting center 1 (IC1) hypermethylation (0.73-0.8). The IC1 (*H19* gene) hypermethylation of chromosome 11p15 increases the risk for Wilms tumor due to the biallelic expression of the *IGF2* gene (34). The IC1 hypermethylation has been observed in 5–10% of Beckwith-Wiedemann patients and among the molecular subgroups of BWS represents an increased risk to develop a malignancy of a kidney (Wilms tumor) or liver (hepatoblastoma) (35). Although the cnnLOH analysis from ES data indicates the cnnLOH of chromosome 11p15p13, mosaic IC2 (*KCNQ1OT1*) hypomethylation using the MS-MLPA analysis was not detected most likely due to the inability of MS-MLPA to identify low-level mosaic imprinting defects (36). Most individuals affected by BWS or SRS are affected by mosaic imbalances of IC1 and IC2 on chromosome 11p15 (37). The borderline mosaic cnnLOH 11p may be a result of a defective homologous recombination due to the aberrant double-strand break repair caused by the dysfunction of the BLM RecQ-like helicase. The increased frequency of SCE events and mosaic cnnLOH are typical markers of Bloom syndrome (22,38).

The initial clinical diagnosis of our proband was SRS due to the severe prenatal and postnatal growth restriction. The microarray CGH+SNP array and BWS/RSS MS-MLPA excluded that diagnosis. The subsequent analysis using trio-based ES elucidated the diagnosis of Bloom syndrome. As well as previously documented in some cases of Bloom syndrome, some of the cases do not manifest typical features, such as sun-induced, butterfly-shaped skin lesions, which would have led to a clinical misdiagnosis (27,39). The mosaicism for IC1 (*H19* gene) hypermethylation and differences of has been observed in a subset of patients with BWS in a risk for embryonal tumors in early childhood (40). The distribution of chromosome 11p15 mosaicism for methylation changes can significantly vary between tissues, so additional tissue-specific testing may be valuable in personalized medical intervention (41).

A diagnosis of Bloom syndrome carries a greatly increased risk to develop early-onset malignancies and then an increased life-time risk to develop multiple malignancies due to the genome instability. Therefore, the co-existence of cancer-predisposing Bloom syndrome and risk factors resulting from the IC1 11p15 hypermethylation due to the mosaic cnnLOH of chromosome 11p could classify our proband as a highly-risk individual requiring the multidisciplinary medical and therapeutic observation and prospective medical intervention (42).

A rapid molecular genetic diagnostics using trio-based ES for the simultaneous detection of sequence variants, CNVs and cnnLOH improves the quality of medical care due to the early medical surveillance, interventions and optimal setting of a specialized healthcare of pediatric patients with rare diseases with an adverse prognosis.

## Supplementary Material

Supporting Data

Supporting Data

Supporting Data

Supporting Data

## Data Availability

The datasets generated for the present case report, including raw and processed ES outputs of proband and her parents, microarray analysis and qPCR are not publicly available due to the protection of individuals' privacy, but are available from the corresponding author on reasonable request. The full visualization of presented BLM gene variants (IGV software v2.8.13), Sanger sequencing data, including the chromatograms of the proband, her parents and their relatives for the BLM pathogenic variants and the MS-MLPA outputs for the borderline H19 hypermethylation in the proband are stored in the Figshare online digital repository under DOI: doi.org/10.6084/m9.figshare.19727653.

## References

[1] Posey JE (2019). Genome sequencing and implications for rare disorders. Orphanet J Rare Dis.

[2] Cummings BB, Marshall JL, Tukiainen T, Lek M, Donkervoot S, Foley AR, Bolduc V, Waddell LB, Sandaradura SA, O'Grady GL (2017). Improving genetic diagnosis in Mendelian disease with transcriptome sequencing. Sci Transl Med.

[3] Patel DS, Misenko SM, Her J, Bunting SF (2017). BLM helicase regulates DNA repair by counteracting RAD51 loading at DNA double-strand break sites. J Cell Biol.

[4] Cunniff C, Bassetti JA, Ellis NA (2017). Bloom's syndrome: Clinical spectrum, molecular pathogenesis, and cancer predisposition. Mol Syndromol.

[5] Traverso G, Bettergowda C, Kraus J, Speicher MR, Kinzler KW, Vogelstein B, Lengauer C (2003). Hyper-recombination and genetic instability in BLM-deficient epithelial cells. Cancer Res.

[6] Vojta V (1965). Rehabilitation des spastischen infantilen syndroms. Eigene Methodik. Orthop Traumat.

[7] Howe B, Umrigar A, Tsien F (2014). Chromosome preparation from cultured cells. J Vis Exp.

[8] Livak KJ, Schmittgen TD (2001). Analysis of relative gene expression data using real-time quantitative PCR and the 2(−Delta Delta-C(T)) method. Methods.

[9] Wayhelova M, Vallova V, Broz P, Mikulasova A, Loubalova D, Filkova H, Smetana J, Drabova K, Gaillyova R, Kuglik P (2022). Novel de novo pathogenic variant in the GNAI1 gene as a cause of severe disorders of intellectual development. J Hum Genet.

[10] Chen S, Zhou Y, Chen Y, Gu J (2018). fastp: An ultra-fast-all-in-one FASTQ preprocessor. Bioinformatics.

[11] Li H, Durbin R (2009). Fast and accurate short read alignment with Burrows-Wheeler transform. Bioinformatics.

[12] Koboldt DC, Chen K, Wylie T, Larson DE, McLellan MD, Mardis ER, Weinstock GM, Wilson RK, Ding L (2009). VarScan: Variant detection in massively parallel sequencing of individual and pooled samples. Bioinformatics.

[13] R Core Team (2020) R: A language and environment for statistical computing.

[14] McLaren W, Gil L, Hunt SE, Riat HS, Ritchie GRS, Thormann A, Flicek P, Cunningham F (2016). The ensembl variant effect predictor. Genome Biol.

[15] Landrum MJ, Lee JM, Benson B, Brown GR, Chao C, Chitipiralla S, Gu B, Hart J, Hoffman D, Jang W (2018). ClinVar: Improving access to variant interpretations and supporting evidence. Nucleic Acids Res.

[16] Kopanos C, Tsiolkas V, Kouris A, Chapple CE, Aguillera MA, Meyer R, Massouras A (2019). VarSome: The human genomic variant search engine. Bioinformatics.

[17] Hamosh A, Scott AF, Amberger JS, Bocchini CA, McKusick VA (2005). Online mendelian inheritance in man (OMIM), a knowledgebase of human genes and genetic disorders. Nucleic Acids Res.

[18] Lappalainen I, Lopez J, Skipper L, Hefferson T, Spalding JD, Garner J, Chen C, Maguire M, Corberr M, Zhou G (2013). DbVar and DGVa: Public archives for genomic structural variation. Nucleic Acids Res.

[19] Kendall KM, Bracher-Smith M, Fitzpatrick H, Lynham A, Rees E, Escott-Price V, Owen MJ, O'Donovan MC, Walters JTR, Kirov G (2019). Cognitive performance and functional outcomes of pathogenic copy number variants: Analysis of the UK Biobank. Br J Psychiatry.

[20] Miller DT, Lee K, Abul-Husn NS, Amendola LM, Brothers K, Chung WK, Gollob MH, Gordon AS, Harrison SM, Hershberger RE (2022). ACMG SF v3.1 list for reporting of secondary findings in clinical exome and genome sequencing: A policy statement of the American College of medical genetics and genomics (ACMG). Genet Med.

[21] Li L, Eng C, Desnick RJ, German J, Ellis NA (1998). Carrier frequency of the Bloom syndrome blmAsh mutation in the Ashkenazi Jewish population. Mol Genet Metab.

[22] Montenegro MM, Quaio CR, Palmeira P, Gasparini Y, Rangel-Santos A, Damasceno J, Novak EM, Gimenez TM, Yamamoto GL, Ronjo RS (2020). Gene expression profile suggesting immunological dysregulation in two Brazilian Bloom's syndrome cases. Mol Genet Genomic Med.

[23] German J, Sanz MM, Ciocci S, Ye TZ, Ellis NA (2007). Syndrome-causing mutations of the BLM gene in persons in the Bloom's syndrome registry. Hum Mutat.

[24] Gönenc, Elcioglu NH, Grijalva CM, Aras S, Großmann N, Praulich I, Altmüller J, Kaulfuß S, Li Y, Nürnberg P (2022). Phenotypic spectrum of BLM- and RMI1-related Bloom syndrome. Clin Genet.

[25] Backers L, Parton B, De Bruyne M, Tavernier SJ, Van Den Bogaert K, Lambrecht BN, Haerynck F, Claes KBM (2021). Missing heritability in Bloom syndrome: First report of a deep intronic variant leading to pseudo-exon activation in the BLM gene. Clin Genet.

[26] Gönenc, Wolff A, Schmidt J, Zibat A, Müller C, Cyganek L, Argyriou L, Räschle M, Yigit G, Wollnik B (2022). Single-cell transcription profiles in Bloom syndrome patients link BLM deficiency with altered condensin complex expression signatures. Hum Mol Genet.

[27] Suspitsin EN, Sibgatullina FI, Lyazina LV, Imyanitov EN (2017). First two cases of Bloom syndrome in Russia: Lack of skin manifestation in a BLM c.1642C>T (p.Q548X) homozygote as a likely cause of underdiagnosis. Mol Syndromol.

[28] Trizuljak J, Petruchová T, Blaháková I, Vrzalová Z, Hořínová V, Doubková M, Michalka J, Mayer J, Pospíšilová Š, Doubek M (2020). Diagnosis of Bloom syndrome in a patient with short stature, recurrence of malignant lymphoma, and consanguineous origin. Mol Syndromol.

[29] Wan L, Han J, Liu T, Dong S, Xie F, Chen H, Huang J (2013). Scaffolding protein SPIDR/KIAA0146 connects the Bloom syndrome helicase with homologous recombination repair. Proc Natl Acad Sci USA.

[30] Sokolenko AP, Iyevleva AG, Preobrazhenskaya EV, Mitiushkina NV, Abysheva SN, Suspitsin EN, Kuligina ES, Gorodnova TV, Pfeifer W, Togo AV (2012). High prevalence and breast cancer predisposing role of the BLM c.1642C>T (Q548X) mutation in Russia. Int J Cancer.

[31] Prokofyeva D, Bogdanova N, Dubrowinskaja N, Bermisheva M, Takhirova Z, Antonenkova N, Turmanov N, Datsyuk I, Gantsev S, Christiansen H (2013). Nonsense mutation p.Q548X in BLM, the gene mutated in Bloom's syndrome, is associated with breast cancer in Slavic population. Breast Cancer Res Treat.

[32] Shahrabani-Gargir L, Shomrat R, Yaron Y, Orr-Urteger A, Groden J, Legum C (1998). High frequency of a common Bloom syndrome Ashkenazi mutation among Jews of polish origin. Genet Test.

[33] Huson SM, Staab T, Pereira M, Ward H, Paredes R, Evans DG, Baumhoer D, O'Sullivan J, Cheesman E, Schindler D, Meyer S (2022). Infantile fibrosarcoma with TPM3-NTRK1 fusion in a boy with Bloom syndrome. Fam Cancer.

[34] Fiala EM, Ortiz MV, Kennedy JA, Glodzik D, Fleischut MH, Duffy KA, Hathaway ER, Heaton T, Gerstle JT, Steinherz P (2020). 11p15.5 epimutations in children with Wilms tumor and hepatoblastoma detected in peripheral blood. Cancer.

[35] Ibrahim A, Kirby G, Hardy C, Dias RP, Tee L, Lim D, Berg J, MacDonald F, Nightingale P, Maher ER (2014). Methylation analysis and diagnostics of Beckwith-Wiedemann syndrome in 1,000 subjects. Clin Epigenetics.

[36] Van Veghel-Plandsoen MM, Wouters CH, Kromosoeto JNR, den Ridder-Klünnen MC, Halley DJJ, van den Ouweland AMW (2011). Multiplex ligation-depending probe amplification is not suitable for detection of low-grade mosaicism. Eur J Hum Genet.

[37] Brioude F, Kalish JM, Mussa A, Foster AC, Bliek J, Ferrero GB, Boonen SE, Cole T, Baker R, Bertoletti M (2018). Expert consensus document: Clinical and molecular diagnosis, screening and management of Beckwith-Wiedemann syndrome: An international consensus statement. Nat Rev Endocrinol.

[38] LaRocque JR, Stark JM, Oh J, Bojilova E, Yusa K, Horie K, Takeda J, Jasin M (2011). Interhomolog recombination and loss of heterozygosity in wild-type and Bloom syndrome helicase (BLM)-deficient mammalian cells. Proc Natl Acad Sci USA.

[39] Bouman A, van Koningsbruggen S, Karakullukcu MB, Schreuder WH, Lakeman P (2018). Bloom syndrome does not always present with sun-sensitive facial erythema. Eur J Med Genet.

[40] MacFarland SP, Duffy KA, Bhatti TR, Bagatell R, Balamuth NJ, Brodeur GM, Ganguly A, Mattei PA, Surrey LF, Balis FM, Kalish JM (2018). Diagnosis of beckwith-wiedemann syndrome in children presenting with wilms tumor. Pediatr Blood Cancer.

[41] Alders M, Maas SM, Kadouch DJM, var der Lip K, Bliek J, van der Horst CMAM, Mannens MMAM (2014). Methylation analysis in tongue tissue of BWS patients identifies the (EPI)genetic cause in 3 patients with normal methylation levels in blood. Eur J Med Genet.

[42] Campbell MB, Campbell WC, Rogers J, Rogers N, Rogers Z, van den Hurk AM, Webb A, Webb T, Zaslaw P (2018). Bloom syndrome: Research and data priorities for the development of precision medicine as identified by some affected families. Cold Spring Harb Mol Case Stud.

[43] Robinson JT, Thorvaldsdóttir H, Winckler W, Guttman M, Lander ES, Getz G, Mesirov JP (2011). Integrative Genomics Viewer. Nat Biotechnol.

