# Supplementary material for: A unique case of Bloom syndrome with a combination of genetic hits: A lesson from trio‑based exome sequencing: A case report
Source: Mol Med Rep. 2023 Apr 12;27(5):110. doi: 10.3892/mmr.2023.12997 (PMC10119849; doi:10.3892/mmr.2023.12997)

Figure S1. Familial 15q11.2 microduplication detected by ES. The panel shows profiles of chromosome 15 in mother, father and proband (from up to bottom). Log2 Ratio (L2R) tracks represent the copy-number profiles. Grey dots mark sequencing targets without any copy-number abnormality. Red and green dots indicate the presence of losses and gains, respectively. In the AF track, black dots represent SNPs with a single allele detected in the sequencing reads and green and red dots highlight SNPs with two alleles found in an approximate 1:1 and another ratio, respectively. The 15q11.2 microduplication in proband and her father is visualized as a group of green dots in the L2R track. ES, exome sequencing; AF, allele frequency; SNPs, single nucleotide polymorphisms.

FAMILY • GAIN • 15q11.2 • chr15:22460670-23130822 • 670153bp

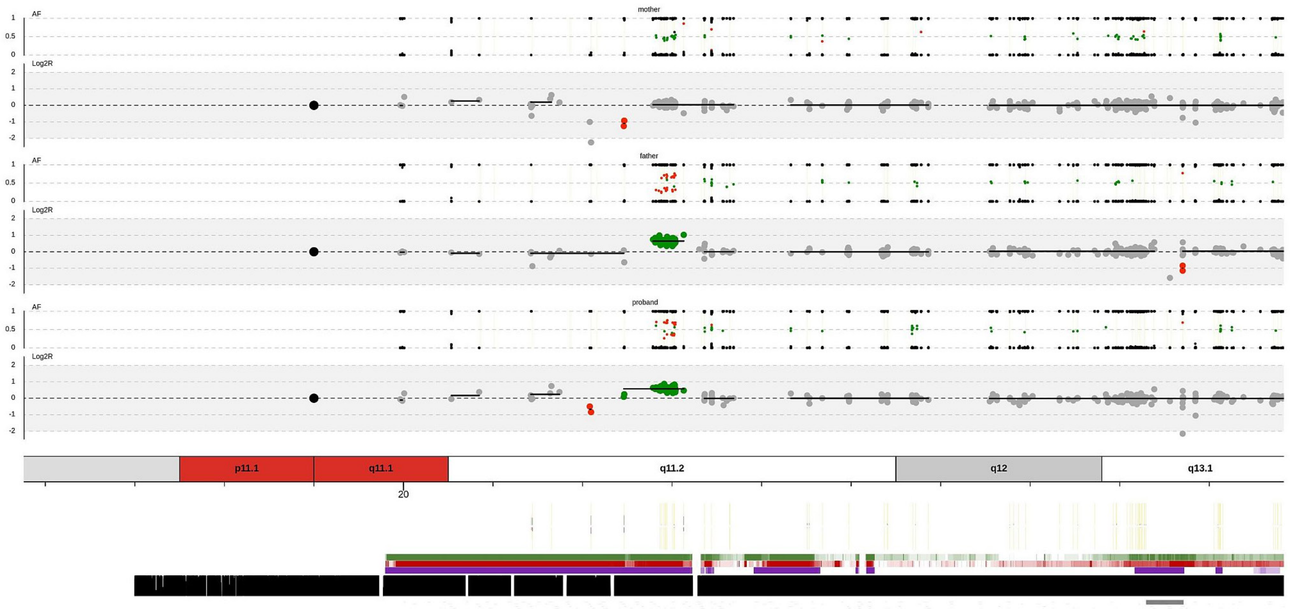

Supplement: Supporting Data [file Supplementary_Data1.pdf]
